# Selected Office Based Anticancer Treatment Strategies

**DOI:** 10.1155/2019/7462513

**Published:** 2019-01-15

**Authors:** Jesse A. Stoff

**Affiliations:** Integrative Medicine of New York, 265 Post Ave., Suite 380, Westbury, NY 11590, USA

## Abstract

Over the years, the treatment of patients with cancer has varied widely as much because of recent advancements in science and medicine as the philosophies that belie their use. This paper briefly describes many of the prevailing approaches in use today with an attempt to offer some perspective of how to apply these disparate methodologies so that they may be more effectively integrated, resulting in consistently better clinical responses.

## 1. Introduction

From a perspective of 10,000 feet there are two basic characteristics of cancer: genetic instability and the propensity to metastasize. From those two basic observational characteristics, the hallmarks of cancer were derived by Drs. Hanahan and Weinberg in 2000 as follows:

  Self-sufficiency in growth signals
  Insensitivity to antigrowth signals
  Evading apoptosis
  Limitless replicative potential
  Sustained angiogenesis
  Tissue invasion and metastasis.

And then the following were added in 2011:

  Deregulating cellular energetics
  Avoiding immune destruction
  Genome instability and mutation
  Tumor-promoting inflammation [1, 2].

Based upon the two basic characteristics of cancer, there are nonspecific medicaments that should be considered in all cases to help stabilize the mutated genetics. Laboratory testing will clarify the ongoing need for these agents and others in their class. As a starting point, they are a safe and efficacious place to begin therapy. The other hallmarks of cancer will be addressed with the strategies that follow.

## 2. Genetic Stabilizers

As a starting point to help my patients who are fighting cancer, there are a few supplements one can use until lab tests and genetic studies are completed. Once the tests and studies are finalized and returned, a more specific treatment strategy can be formulated to address the above hallmarks. Each of these supplements has a multitude of beneficial effects, but chief among them is their ability to stabilize genetics, creating the foundation of an integrative anticancer strategy. During the course of oncogenesis and tumor progression, cancer cells constitutively upregulate signaling pathways relevant to cell proliferation as a result of any number of genetic mutations. It is that genetic instability that leads to cancer being cancer in the first place and it is those mutations which account for the multiple cell lines that compose every tumor.

First on our list of genetic stabilizers is vitamin D. In addition to enhancing DNA repair, vitamin D also induces growth arrest and apoptosis of tumor cells and their nonneoplastic progenitors. Cell-based studies show that the active metabolite 1,25 dihydroxyvitamin D is the biologically active form that works through the vitamin D receptor to regulate gene transcription. Vitamin D (D_3_) is produced from 7-dehydrocholesterol when skin is directly exposed to UVB light which, in more northern locations, is largely filtered out by the atmosphere. Vitamin D is readily sourced from various foods including fish, eggs, caviar (for the gourmets among us), some mushrooms, beef liver, and cheese. Regardless of whether vitamin D comes from the skin or the diet, vitamin D_3_ is transported through the blood by the vitamin D Binding Protein (DBP). Once delivered to the liver, vitamin D is hydroxylated on its side chain to form 25 hydroxyvitamin D (25OH D). This is a stable metabolite whose serum levels are commonly used to assess vitamin D status. As needed and if available, D_3_ circulates to the kidneys which is the primary site where the active form of vitamin D, 1,25(OH)_2_ D, is produced through the genomic actions of 1*α*,25(OH)_2_ via the vitamin D receptor (VDR), and its analogs inhibit cell cycle progression and tumor cell growth. Mechanisms of action range from preventing cell proliferation (cell cycle arrest) in cancer cells to inducing apoptosis or suppressing cell adhesion molecules and growth factors that promote cellular homing and metastasis. It also affords important antioxidant protection and serves as an immunomodulatory for both the innate and adaptive arms of the immune system [3–7].

Next comes indole-3-carbinol and its metabolite 3,3′-diindolylmethane (DIM). They target multiple aspects of cancer cell cycle regulation and survival including Akt-NF*κ*B signaling, caspase activation, and cyclin-dependent kinase activities, stabilize estrogen metabolism, normalize estrogen receptor signaling, reduce endoplasmic reticulum stress, and limit BRCA gene expression. DNA hypermethylation is a common feature of cancer genetics. When methylation detox pathways start to fail, certain regions of the genome will accumulate too many methyl groups, such as at CpG promoter regions (segments of the DNA involved in DNA and RNA transcription); this can lead to increased mutagenesis and eventual cancer development. Much research has demonstrated how DIM reduced methylation at 5 CpG promoter regions. For example, in split population studies, mice given TRAMP prostate cancer cells were also given DIM. The mice given the DIM showed a much lower incidence of cancer and metastasis than controls, as well as much higher expression of antioxidant/anticarcinogen protective enzymes NQO1 and NFR2 in prostate tissues [8–14].

Curcumin, a component of turmeric (*Curcuma longa*), is a low molecular weight molecule that has antiproliferative activity and inhibits tumor initiation and propagation through a variety of pathways. It accomplishes this through several epigenetic effects that result in genetic modulation that then changes the expression of several key proteins, some of which are the cysteine-aspartic acid proteases (caspases). Caspases are a family of enzymes that play an essential role in apoptosis, necrosis, and inflammation. Research demonstrates that curcumin activates caspases-3 and -8 but not caspase-9, indicating that the apoptosis induced in cancer cells occurs via a membrane-mediated mechanism. Membrane bound enzymes play other important roles in the perpetuation of cancer cells such as the ECTO-NOX 2 system.

p53 (TP53) is a tumor suppressor gene and is responsible for protecting cells from tumorigenic alterations. Mutational inactivation of p53 is frequently observed in many cancers. Curcumin selectively increases p53 expression during the G2 phase of the cell cycle of carcinoma cells and releases cytochrome *c* from mitochondria, which is an essential requirement for then inducing p53-dependent apoptosis in the cancer cell.

Another protein affected by curcumin is Akt (protein kinase B), a serine/threonine kinase. It is a critical enzyme in signal transduction pathways involved in cell proliferation, apoptosis, and angiogenesis. Curcumin inhibited the phosphorylation of Akt in a dose dependent manner leading to another pathway of apoptosis.

Curcumin also induces the upregulation of carcinogen-detoxifying enzymes, such as glutathione* S*-transferases, has antioxidation effects, and suppresses cyclooxygenase which then reduces the level of inflammation and the stimulation of cancer stem cells. Real time animal model studies have demonstrated that curcumin also decreased the expression of DNA damage response genes, including ATM, ATR, BRCA1, 14-3-3*σ*, DNA-PK, and MGMT; thus, the reduction of a DNA damage response is but a part of the reason for curcumin-induced growth inhibition of cancer cells [14–16].

Sulforaphane (SFN) is an isothiocyanate found in cruciferous vegetables such as broccoli, brussel sprouts, cauliflower, and cabbage. Experimentally, in cell cultures and animal models, SFN was shown to be a highly effective chemoprotective against, carcinogen-induced, and genetic animal cancer models, as well as in xenograft transplant models of cancer. The early research focused on the detoxification ability of SFN to induce phase 2 enzyme pathways. Later studies showed that SFN could cause cancer cells to enter G2/M phase arrest and result in apoptotic cell death, with the latter being evidenced by caspase-mediated cleavage of poly(ADP-ribose) polymerase and increased release of histone-associated DNA fragments from the tumor downstream. Furthermore, it leads to the transcriptional activation of genes including tumor suppressor genes. The effect on cancer genetics is profound and therapeutically beneficial [17–21].

Fish oil rounds out our list of top genetic stabilizing supplements. One of many changes that occur to the cancer cell's biochemistry and genetics is in the production, metabolism, and expression of microRNAs (miRNAs). First discovered in the early 1990s, their importance as a distinct class of biological regulators was not appreciated for another decade. MicroRNAs (miRNAs) are short molecules, just 21–25 nucleotides long, but can have powerful effects on gene expression. More than 2000 miRNAs have been identified including many specific miRNAs that have been found to be associated with diseases states including cancer and the risk of metastasis. The identification of circulating miRNA specific to metastatic cancer presents a unique opportunity for early disease identification and for monitoring disease burden as a circulating biomarker. For example, abnormal activation of some miRNAs found in the blood, let-151a, miR-21, miR-155, miR-,145 miR-18a, and miR-16, as well as tissue specific miRNAs, miR-182, miR-145, miR-21, miR-155/154, miR-203, miR-213, and miR-7, are often found in patients affected by breast cancer. Furthermore, there is a growing body of evidence on the value of miRNAs associated with the development of drug-resistance, suggesting their values, once targeted, as a potential approach to overcoming chemoresistance. miRNAs that are absorbed into our blood stream from GMO foods are currently being studied for their oncogenic potential, as they are normally foreign to human biology. Fish oil can modulate the expression of the miRNAs including significantly reducing the risk of metastasis and has many other anticancer effects [22–27].

Once the basic genetic stabilizers are in place we move on to more specific, integrative, anticancer strategies which are based upon laboratory results, the patient's history, and our clinical experience. Integrative anticancer strategies are based upon the basic observation that, for a patient to develop, a serious cancer multiple systemic abnormalities must be occurring simultaneously: genetically, biochemically, immunologically, and hormonally, which must be addressed from a functional medicine point of view. An effective treatment strategy addresses these issues while applying pressure against the weak points of the cancer, as determined through laboratory and genetic testing, to buy the time necessary to reconstitute an effective immune response that leads to a durable remission. Assuming that the physician has a good understanding of immunology and biochemistry and a thorough, if not specialized, knowledge of oncology, then these strategies can be added to their armamentarium in a matter of days or weeks of workshops and additional study. There are a number of anticancer strategies that can be employed; which to use when is a matter of objective data collection balanced by experience. Again, in no particular order, as anyone of them could potentially be the one key strategy needed for a particular patient to go into remission, generally, multiple strategies are needed and are timed to the changing conditions clinically as treatment progresses.

## 3. Differentiation Therapy

Differentiation therapy of cancer is a strategy that goes back to the late 1980s and employs agents that can induce both morphological changes and suppress abnormal proliferation in a time and dose dependent manner. Through mechanisms and pathways known and unknown many substances have been found to have this effect including phenylbutyrate (an antineoplaston), dimethyl sulfoxide (DMSO), all-trans retinoic acid (ARTA), vitamin D, and epigallocatechin gallate (EGCG). The need for cellular differentiation and the reversion back to normal cellular phenotypes is universal across all cancers; thus the use of this class of agents is as important as the use of substances that stabilize genetics. Phenylbutyrate (PB) is an aromatic fatty acid that is converted in vivo to phenylacetate (PA) by *β*-oxidation in liver and kidney mitochondria. The actions of PB as a differentiating agent are primarily related to its activity as an inhibitor of histone deacetylase. Phenylbutyrate can be given by mouth or IV; EGCG and ARTA are, at this point, only available in oral forms. DMSO, on the other hand, seems to be effective only when given IV. Each agent considered must have been shown to be effective for the type of cancer being treated before being included in the therapeutic strategy. Then, depending upon the competence of the patient's digestive system and issues of IV access, appropriate substances can then be used [28–31].

Anticancer strategies that build upon genetic stabilizers, hormone blockade, and differentiation agents do not necessarily put the cancer into a durable remission. They do, however, in many cases slow its rate of growth enough so that the immune system, with support, can catch up to and destroy the tumor(s). Many other strategies are available that may be oncolytic and can be used based upon the results of the patient's biochemical and immune tests as well as a thorough analysis of the intrinsic genetics driving the cancer. The combination of this data allows for an individualized, integrative therapeutic strategy. There is no clear hierarchy or “best answer for cancer”; it depends upon how the patient presents and the targeted therapies available at the time that the cancer's genetics are determined. The “best” strategy to be employed is a function of this information and the current state of the available technology and agents to address it. Some of those strategies are further discussed below:

- Oncolytic virotherapy
- Cytotoxic strategies
- Immunotherapy
- Biochemical treatments
- Targeted therapies
- Antiangiogenesis strategies
- Cancer stem cell suppression
- Oxidative therapies
- Anti-inflammatory strategies
- Detox strategies
- Nutritional diet strategies
- Psychoneuroimmunology
- Targeted genetic strategies
- Hyperthermia.

## 4. Oncolytic Virotherapy

Interest in oncolytic virotherapy, as a treatment strategy, has waxed and waned for decades. For centuries physicians have observed that, in some lucky cases, if patients with cancer, even very advanced cancers, contracted a significant acute illness, bacterial or viral, sometimes the cancer would go into remission. Building upon these observations, Dr. Coley made a scientific assessment of this phenomenon which he published in 1891 in the Annals of Surgery. He then set about to reliably replicate these results by developing a sterile filtrate of bacterial toxins from Strep and Serratia. He gave these toxins to patients with cancer, recorded that in many cases their tumors would indeed go into remission, and published his results in 1893. This first consistently useful immunotherapeutic strategy was then medically usable until 1963 when changes in the structure and politics of the FDA essentially made it unavailable. However, the basic observation that a significant febrile illness (as a sort of internal hyperthermia) could put a cancer into remission did not go away and research began to investigate the use of self-limiting viral infections as a way of conquering cancer. There have been many reports, usually of farmers, who had cancer and then contracted the Newcastle virus from a sick chicken, having their cancer go into remission. But, since the Herpes Simplex virus was better understood, at the time, than the Newcastle virus, it was the first to be used in a clinical trial in 1996. The virus may infect healthy and cancerous cells but normal cells, with their defensive pathways still intact, are able to fight off the infection while the cancerous cell succumb to the apoptotic triggers of the infection. Thus the virus becomes oncolytic. Viruses that cause mild and self-limiting infections in humans are used and modified to accomplish this. Treatment with oncolytic viruses results in a combination of tumor-specific cell lysis together with dynamic immune stimulation, therefore acting as a sort of in situ tumor vaccine. Oncolytic viruses that are currently used are “engineered” for optimization of tumor selectivity and enhanced immune stimulation and can be readily combined with other therapeutic strategies. Their effectiveness has been demonstrated in many preclinical studies and clinical protocols.

A related biological strategy is the use of modified live bacteria called Bacteria Mediated Tumor Therapy (BMTT). The first scientific application of this therapy was with BCG, or bacille Calmette-Guerin, which was developed as a vaccine for tuberculosis (TB) disease and then found to be helpful when instilled into the bladder to fight certain types of bladder cancer. More recent developments include the use of genetically modified salmonella, clostridium, and the plasmids implanted in Listeria. These treatments are thus far less effective because they are designed to present or bind to cancer antigens to make them more immunogenic but they do nothing to address the damaged immune system pathways that allowed the cancer to occur to begin with [32–39].

## 5. Cytotoxic Cancer Therapy

Cytotoxic cancer treatments are covered by the term chemotherapy and have come to imply the use of agents that act as intracellular poisons to inhibit mitosis or directly induce some sort of cell death. Substances that accomplish this as a secondary effect through blocking extracellular signals or which act through a specific genetic, enzymatic, or hormonal pathway are excluded from this therapeutic strategy as they are referred to as targeted therapies and elsewhere described. Chemotherapeutic agents are characteristically purified to the point of being a chemical and may be of natural origin or synthetically created. The deliberate use of natural, herbal source agents began in the early 1920s, including Viscum alkaloids and lectins, and they are still used today, whereas the first synthetic agent, nitrogen mustard, was “discovered accidentally” during World War Two when it was observed that it could shrink lymphoma tumors in mice. A few years later it was discovered that alkaloids extracted from the Vinca rosea plant were useful in treating Hodgkin's disease, and so a multibillion dollar industry was birthed creating and extracting new substances, helpful in the “war on cancer”. Unfortunately, many of these substances have similar effects on healthy cells, thus creating a multitude of side effects and limiting their usefulness. Newer protocols that use lower doses of these agents, such as metronomic or insulin potentiated chemotherapy, are showing good results with far fewer side effects. Metronomic, low dose chemotherapy seems to act through several mechanisms including inhibiting the growth of new blood vessels, the restoration of an anticancer immune response, and the induction of tumor dormancy and has been in development for two decades, whereas Insulin Potentiated Therapy (IPT) takes physiologic advantage of the excessive number of insulin receptors found on the cell surface of cancer cells. Giving insulin just prior to the infusion of low dose chemotherapeutic agents, usually in a combination designed to intervene at several sites of the cell cycle, causes much less side effects and can hold the cancer at bay while buying time to reconstitute an effective immune response or integrate another therapeutic strategy. IPT has been in development and clinical use since the 1930s and has been used to help treat other chronic diseases as well [40–48].

## 6. Immunotherapy

Immunotherapy is an office based strategy for which they write very thick books, and in the broadest terms possible it is a treatment that uses the immune system, in some way, to identify, attack, and destroy the cancer. Using the immune system against cancer can be accomplished through active or passive pathways. As a quick overview, there are over one hundred and eighty-seven cell types identified within the immune system. It includes the lymphatic system along with the bone marrow spleen, thymus, and, of course, lymph nodes. Cells known as CD4 T cells are the “apparent brains” of the immune system and they coordinate the central immune response to any serious health threat. Activated B cells become plasma cells and, in most instances, generate an antibody response against bacterial and viral invasion. Lymphokine activated killer cells and cytotoxic T cells respond to viruses and cancer. Suppressor T cells are used to downregulate the actions of the immune system after the threat has been eliminated through negative feedback. Macrophages are voracious amoeboid-like lymphocytes that eat foreign substances and send a message back to the rest of the immune system indicating what further immune response is necessary. Macrophages and their cousins, the dendritic cells, are involved in all aspects of the immune response by initially sending out the alarm that something is amiss. Natural Killer cells are preprogrammed at their birth to destroy virally infected cells, cancer cells, some bacteria and parasites on contact without need for further direction from the CD4 cells. Some mushroom extracts and the bioengineered nutraceutical AiE10 can increase the concentration and activity of natural killer cells.

The immune response cascade operates according to three directives. The first is to recognize that which is foreign and sound the alarm soon enough to thwart the invader. Molecules and cell surfaces that are identified as foreign are referred to as antigens and have the ability to elicit an immunogenic response. The second directive is to respond to the alarm with enough of a counterattack to effectively neutralize the invader quickly. The third directive is to remember what happened so that if the same situation were to arise again, an effective response could be generated faster. The length and efficacy of the immune response depend upon the “intactness” of the underlying biochemistry. The immune response cascade is the ultimate biological information processing and transfer vehicle designed to define, defend, and integrate oneself relative to the environment that surrounds us. When there is a miscommunication, disease ensues due to corruption, misdirection, or a lack of that informational flow. Immunotherapy is designed to correct, stimulate, direct, or reconstitute an effective anticancer response.

Active immune therapies include, among other agents, IL-2, IFN-g, and IFN-a cytokines to stimulate the TH1 cells, pathways, and natural killer cells. Monoclonal antibodies, checkpoint inhibitors, that disinhibit an immune response are also an active immunotherapy that seems to be most effective when some support has been given to the immune system first. Other active therapies include creating primed dendritic cells (for example, Sipuleucel-T (Provenge)) and natural killer cells and then infusing them into the patient to “patch” holes and reconstitute an effective anticancer response.

Passive immunotherapies include the use of infused antibodies to bind to cancer cells. Then when natural killer cells encounter antibody-coated cells, the latter's Fc regions interact with their Fc receptors, releasing perforin and granzyme B to kill the tumor cell. Adoptive T cell therapy is another passive immunotherapy. Several ways of producing and obtaining tumor targeted T cells have been developed. T cell Infiltrating Lymphocytes (TILs), specific to the tumor antigens, can be removed from a tumor sample with a core biopsy and then purified with a cell separator or filtered from the blood. Subsequent activation with cytokines and cell culturing is performed* ex vivo*, and then the results were reinfused into the patient. Activation can take place through gene therapy or by exposing the T cells to tumor antigens in the presence of cytokines. TILs can also be stimulated in vivo with hypofractionated SBRT to induce an abscopal effect, which when achieved can have miraculous results.

A new, state-of-the-art passive immunotherapy is the use of chimeric antigen receptors (CARs, also known as chimeric immunoreceptors, chimeric T cell receptors, or artificial T cell receptors) which are bioengineered cell receptors that combine a new specificity with an immune cell to target cancer cells. Essentially, what is done is that specific monoclonal antibody fractions are grafted onto T cell receptors. The receptors are referred to as chimeric because they are a fusion of proteins and receptors from different immune sources. CAR-T cell therapy refers to an infusion of such specifically transformed cells for targeted cancer therapy. CAR-T cells destroy the cancer cells through several mechanisms such as having a direct cytotoxic effect and/or stimulating other cells of the immune system through the release of various cytokines and growth factors. Due to the CAR-T cells very narrow specificity, less prominent tumor cell lines can be missed allowing for a future recurrence [49–59].

## 7. Biochemical Anticancer Strategies

Biochemical anticancer treatments, which include the use of off-label legend pharmaceuticals, are numerous and growing but a few of them are briefly reviewed here. Aside from herbal therapies that stretch back millennia, vitamin C may be the most researched and longest used of the biochemical strategies. Historically, in 1954, Dr. McCormick observed that patients with cancer did better when supplemented with vitamin C and wrote that he believed that this was accomplished by increasing collagen synthesis in healthy tissue so that cancer was less able to spread. Later, in 1972 Drs. Cameron and Rotman hypothesized that vitamin C could have anticancer action by inhibiting hyaluronidase and thereby preventing cancer spread, and this hypothesis was further supported by Drs. Cameron and Pauling. They then published clinical research results of 100 patients with terminal cancer, in whom conventional therapy was no longer considered useful, and who were treated with 10 g of vitamin C intravenously for 10 days followed by 10 g orally indefinitely. The patients who received the vitamin C were compared to 1,000 retrospective controls who had similar disease diagnosis and stage, but who did not receive vitamin C or any other definitive anticancer therapy. Those who received vitamin C survived 300 days longer than those who did not receive vitamin C. Many other studies have since been done, with more sophisticated strategies and better long term results of progression free survival, overall survival, and improvements in the quality of life.

Further research on how vitamin C can be cytotoxic to cancer cells was first based upon studying the enzymes responsible for cell death. Low levels of alkaline and acid DNase constitute a characteristic of all nonnecrotic cancer cells in animals and humans. It was found that these enzymes are reactivated at early stages of cancer cell death by vitamin C (acid DNase) in combination with vitamin K(3) (alkaline DNase). Specifically, the coadministration of these vitamins (in an optimal ratio of 100:1, for C and K(3), respectively) produced selective cancer cell death without harm to normal cells and tissues. Pathology studies indicated that cell death is produced mainly by a newly discovered process called autoschizis. Several mechanisms have been described as leading to such a cell death induced by vitamins C and K3 (CK(3)), which included formation of H(2)O(2) during vitamins redox cycling, oxidative stress, DNA fragmentation, no caspase-3 activation, and cell membrane injury with progressive loss of organelle-free cytoplasm as a result of oxidative damage.

In the late 1920s Dr. Otto Warburg was performing ground breaking research on cellular respiration, including that of cancer cells. He found significant and important differences between normal and cancer cells noting that their metabolism was largely based on an anaerobic fermentation metabolism due to damaged, malfunctioning mitochondria. This brilliant insight opened the door for several biochemically based anticancer strategies. As a result of unstable genetics and an inefficient metabolism, cancer cells often die almost as fast as they multiply accounting for the release of tumor markers into the blood stream and allowing for still other anticancer strategies such as antiangiogenesis to be utilized. Since the cells are already unstable, sometimes a little push in the right direction is enough to finish them off. One such strategy is the use of *α*-lipoic acid and hydroxycitrate administered IV and PO, respectively. Two enzymes that are commonly altered during carcinogenesis are pyruvate dehydrogenase (PDH), which is downregulated, and ATP citrate lyase, which is overexpressed. Alpha lipoic acid is a cofactor of PDH, while hydroxycitrate is a known inhibitor of ATP citrate lyase. When hydroxycitrate and *α*-lipoic acid are given together, experimentally in cell cultures, a significant cytotoxic effect was observed: complete cell death was seen following 8 microM lipoic acid and 300 microM hydroxycitrate treatment for 72 h. The combination of alpha lipoic acid and hydroxycitrate was then administered to healthy mice, at doses currently utilized for other indications than cancer; no demonstrable toxicity was observed. In murine cancer models the combination of these two agents was found to be very effective against bladder carcinoma, lung cancer, melanoma, and several other cancers with efficacy at least similar to conventional combination chemotherapy, without the toxicity. However, a major limitation to using this combination of *α*-lipoic acid and hydroxycitrate is that they can only be effective if the mitochondria are still present and/or functional, which is not often the case in the most aggressive tumors. The increased intracellular alkalosis found in most tumors is a strong mitogenic signal, which is resistant to most inhibitory signals. Simultaneous correction of this alkalosis may prove to be necessary for this strategy to be consistently effective on its own. However, studies have demonstrated that integrating these two agents with chemotherapy can give improved short term and long term results.

ECTO (because of their location on the cell's surface) -NOX proteins comprise a family of NAD(P)H oxidases that exhibit both oxidative and protein disulfide isomerase-like activities. The activity of the NOX enzymes correlates with rate of cell growth as they are an important pathway for energy production, which helps to determine how rapidly cells will divide. Normally, when NOX enzyme activity is inhibited, cells fail to enlarge following division and the result is a population of small cells unable to reach the minimum size required for them to divide again. Tumor-associated NOX (tNOX) are novel cell surface ECTO-NOX proteins that are critical for the growth and activity of cancer cells whose metabolism is dependent on a fermentation process for energy production; thus, tNOX, has been identified as a target for cell killing (apoptosis) of cancer cells. A tumor-associated NOX (tNOX) is unregulated, refractory to hormonal regulation and growth factor suppression, but responds to quinone-site inhibitors. Research has demonstrated that among the most potent and effective inhibitors of tNOX are naturally occurring polyphenols exemplified by the principal green tea catechin (-)-epigallocatechin gallate (EGCG), the vanilloid capsaicin, and the chemotherapy agent doxorubicin hydrochloride (Adriamycin).

There are other biochemically based protocols, but intervening in these 3 critical pathways are examples of potent clinical therapies utilizing CK(3), alpha lipoic acid and hydroxycitrate, and EGCG and capsaicin, respectively. These strategies have proven to be safe and effective in vitro, in animal models and in small scale human trials, and should be considered as important allies on the war against cancer [60–84].

## 8. Targeted Therapies

Targeted therapies against cancer fall into several classes of therapeutic agents that act through a variety of direct and indirect effects. Direct approaches target tumor proteins (antigens), enzymes, or genes to alter their signaling, information transduction, or expression. Examples of direct acting agents include monoclonal antibodies (MoAbs), metalloproteinase inhibitors, tyrosine kinase inhibitors, and angiogenesis inhibitors. Indirect acting agents include hormone receptor blockers and epigenetic modulators.

The first monoclonal antibody approved for clinical use was Rituximab in 1997 as a specific therapy for B-cell lymphomas. Since then it has proven effective for several other diseases. The use of monoclonal antibodies is currently the most rapidly growing therapeutic strategy in oncology and has proven to be remarkably effective with less side effects than the chemotherapeutic agents that they are replacing.

Matrix metalloproteinases (MMPs) inhibitors downregulate or block enzymes that break down connective tissue and allow cancers to invade local tissues and metastasize and potentiate angiogenesis. They are a family of zinc-dependent endopeptidases that are a driving factor for the progression of cancer and, thus, their activity has a direct bearing on patient prognosis. One of the simplest and earliest MMPs is EDTA which has been shown to also be helpful in slowing or stopping the progression of cardiovascular heart disease.

Tyrosine kinase inhibitors block an enzyme that functions to transfer a phosphate group from ATP to a cellular protein and acts as an “on”/“off” switch for many cellular functions and the expression of various genes that effect cancer. Phosphorylation of proteins by (tyrosine) kinases is an important mechanism in communicating signals within a cell (signal transduction) and regulating cellular activity, such as (un)regulated cell division. They can become mutated and “stuck” in the “off” position and allow unregulated growth of the cell, such as in the case of p53.

Hormone therapy for cancer is one of the major modalities of targeted therapies used against hormone receptor positive cancers and involves the manipulation of some aspects of the endocrine system through the administration of exogenous agents. These medications may inhibit the production of specific hormones or act to block their receptor sites and in doing so will affect the expression of various genes in cancer cells causing their growth to slow, stop, or trigger an apoptotic cycle. Some of these agents include aromatase inhibitors, gonadotropin-releasing hormone (GnRH) analogs that suppress hormone production, hormone receptor antagonists, selective estrogen receptor modulators (SERMs), antiandrogens, progestogens, and somatostatin analogs [85–92].

## 9. Antiangiogenesis Strategies

Antiangiogenesis is often critically important in slowing the growth of cancer. For tumors to enlarge, they need an increased blood supply to deliver the nutrients needed to produce new cells. Over 60% of tumors create and release vascular endothelial growth factor (VEGF) which is a key pathway for the induction and growth of new blood vessels. Some of the principle stimuli for this production are an anoxic environment, inflammatory molecules, and oncogenic mutations. Once stimulated, the angiogenic switch leads to the cancer cell's expression of proangiogenic factors that increase the tumor's vascularization such as angiogenin, VEGF, fibroblast growth factor (FGF), and transforming growth factor-*β* (TGF-*β*). Agents that inhibit this response range from ammonium tetrathiomolybdate (ATM), which chelates the copper that these enzymes need as a cofactor, to EGCG, monoclonal antibodies, and genetically modified bacteria, among other things. Research in this area is very important and promising as it affects a key pathway needed for the progression of cancer as clearly demonstrated by 4D ultrasound [93–97].

## 10. Cancer Stem Cell Suppression

Cancer stem cells (CSCs) mediate tumor initiation and progression, and inhibiting them is an emerging new area of research to prevent, stop, and reverse cancer. Oncologic research has established that subpopulations of cells identified by monoclonal antibodies to specific cell surface markers behaved like developmental stem cells in their capacity to regrow the human tumors for multiple generations in experimental immune-deficient hosts. In all of the cancers studied so far, there is good evidence that CSCs are relatively resistant to radiation therapy and chemotherapy indicating that novel CSC-targeted therapies are needed. Several pathways are promising targets against CSCs including inducing their apoptosis, inhibiting stem cell self-renewal to either stop their division or to promote their differentiation, or targeting the CSC milieu that supports them. The anti-CSC agents are categorized under two broad headings: small- and macromolecules with different subclasses such as kinase inhibitors and polypeptides. One of the first and safest agents to be recognized as a CSC inhibitor is Metformin [98–110].

## 11. Oxidative Therapies

Oxidative therapies can be an important adjunctive therapy because tumor hypoxia is an adverse factor for a useful clinical response to chemotherapy and radiotherapy and stimulates the activity of cancer stem cells. Oxidative therapies are the subject of research and clinical trials. An example of an oxidative therapy that directly increases, at least, blood oxygen levels is intravenous ozone therapy. Biochemically CoQ10 has been shown to increase oxygen tension in the blood. The effects of both of these and other agents against cancer have been shown to be helpful in a number of clinical trials but many of the details are yet to be worked out [111–117].

## 12. Anti-Inflammatory Strategies

Anti-inflammatory strategies are of critical importance against cancer. In 1498 Albrecht Durer created the famous woodcut entitled “The Four Horsemen, from the Apocalypse” which, in a sense, foreshadowed the epidemic of severe and chronic diseases that we now face. From a biochemical, thousand-foot perspective there are four major processes that are common pathways for the initiation and promotion of cancer. They should be assessed for each patient: glycation can be measured with blood sugar, hemoglobin A1C, and GlycoMark; methylation can be watched with homocysteine levels; oxidation is reflected in levels of lipid peroxides and oxidized LDL; finally we come to inflammation for which there are a myriad of markers including ESR, CRP, plasma viscosity, adiponectin, monocyte chemoattractant protein 1 (MCP-1), CD40 ligand and lipoprotein-associated phospholipase A(2) (Lp-PLA(2)), and ferritin to name but a few. Inflammation results from or is triggered by virtually every disease known to man and has cellular pathways, plasma cascades, and acute and chronic markers. Some the markers associated with cancer include CRP, IL-6, IL-8, and TNF-alpha. Inflammation can stimulate cancer stem cells as well as more mature tumor cells triggering progression. Inflammation is a defense mechanism that serves to protect us from the illnesses and injuries that historically, from an evolutionary point of view, were the main causes of death. Inflammatory markers activate the Th2 pathway which generates more inflammation. In doing so, in an apparent effort to focus and conserve resources, when the Th2 pathway becomes chronically stimulated by infection, nutritional deficiency, trauma, stress, or toxins, it suppresses the Th1 pathway which is the critical pathway of the immune system needed to protect us from and fight cancer.

An important enzyme for the inflammatory response is cyclooxygenase (COX), which is critical for the conversion of arachidonic acid to prostaglandins and other Eicosanoids. It exists as two isoforms: COX-1 and COX-2; COX-1 is constitutively expressed whereas COX-2 is a highly inducible gene that is activated by cytokines, growth factors, phorbol esters, oncogens, and chemical carcinogens. COX-2 plays a key role in carcinogenesis as has been demonstrated in numerous cancer cell types. Multiple pathways have been proposed to explain how increased COX-2 expression might contribute to carcinogenesis including elevated BCL-2 protein levels and inhibition of apoptosis, increased angiogenesis, and enhanced metastatic activity. Suppressing COX-2 activity, or at least not stimulating it, which many chemotherapeutic agents do, is an important part of any anticancer strategy. Many agents have been identified that suppress COX-2 activity, which can be assayed in vitro and is reflected clinically in decreased levels of CRP, interleukin-6 (IL-6), P-selectin, matrix metalloproteinase-9 (MMP-9), tumor necrosis factor (TNF-*α*), and other inflammatory markers. Some of the anti-inflammatory agents that are helpful against cancer include Celecoxib, fish oil, eicosapentaenoic acid (EPA) and docosahexaenoic acid (DHA), white willow tree, curcumin, green tea, pycnogenol, boswellia, resveratrol, cats claw, and capsaicin to name just a few of the most common medicaments [106–128].

## 13. Detox Strategies

Detox strategies have been used against cancer since time immemorial. When confronted with cancer, people have always (rightfully so) felt the need to “detoxify”. In the past, family history was a key factor in assessing ones risk for getting cancer. Now, most cancers are environmentally induced with the inducers numbering in the thousands but, by the time that a cancer appears from them, a simple detox strategy is not usually enough to stop and reverse it. However, studies have shown that removing, chelating, or neutralizing toxins is still helpful in fighting the good fight as most of them are shown to initiate or promote cancer through a number of pathways including being proinflammatory, hormonal deregulators, and immunosuppressants, or cause direct or indirect DNA damage. No one strategy has been shown to be magical but they range from water fasts to Essiac tea, Hoxsey herbal formulas, Gerson diet, Paleo organic diets, and ketogenic diets to name but a few. The common denominators seem to be a low carbohydrate load, organic and plenty of filtered water. Other office based detox strategies include specialized massage therapies and Ayurvedic Panchakarma, but to be effective they require years of training [129–131].

## 14. Nutritional Diet Strategies

Nutritional diet strategies are usually closely related to detox strategies like two sides of the same coin. Whereas detox seeks to remove something from the body, nutritional strategies add specific nutrients or agents to make up for a deficiency or stimulate an enzyme pathway. Clinical deficiencies can be absolute or relative. An absolute nutritional deficiency occurs when someone is not consuming a diet with the needed nutrient(s) or there is a malabsorptive issue with their digestive tract. A relative nutritional deficiency occurs when they are consuming the appropriate nutrients but they are “burning” them faster than they are absorbing them. For example, vitamin C requirements increase as the body is under higher levels of stress: mental, emotional, or physical. If there are nutritional deficiencies, then the underlying enzymes will falter, and since the basis of a functional immune system is an intact biochemistry, an effective immune response will fail. Doing blood tests for nutrients is thus very important at the onset of treatment and periodically throughout its course [132–136].

## 15. Psychoneuroimmunology

Psychoneuroimmunology is new field of medical practice that takes advantage of the observation that the (neuro)endocrine system plays a critical role in resurrecting immune defenses. The issue here is one of stress and its medical counterpart, psychoneuroimmunology (PNI). In a nutshell, PNI is a scientific field of study that views the immune system as capable of behaving like a bidirectional sensory organ and, therefore, to some degree, under our conscious control and ultimately the key to the mind-body connection. The powerful healing effects that can occur when the mind is engaged in the battle against cancer have been well documented for over thirty years in over 100 books and 9,000+ medical journal articles.

Well documented research began in the 1960s when Dr. George Solomon discovered that by electrically stimulating certain parts of an animal's brain, its ability to fight infection could be improved. By damaging those same parts, its immune function could be impaired. Seeing some indications that macrophages and other defensive cells had specific binding sites for neurotransmitters (including the mood-altering endorphins) on their outer membranes, Solomon and many of his contemporaries began to suspect that feedback from the immune system might even affect the emotional and rational centers of the brain. This would explain, in part, why people get irritable when they are sick and why mental capacity (like in dementia) often deteriorates in parallel with resistance to disease.

Many recent studies have clearly shown this field to be analytically scientific, for example, PMID: 15220929. This study showed the following:

“Thus we show that an epigenomic state of a gene can be established through behavioral programming, and it is potentially reversible.” [137–140]

## 16. Targeted Genetic Strategies

New cancer genetic breakthroughs are being made nearly on a daily basis. These new discoveries demonstrate the increasingly important role of biomarker analysis and tumor profiling, and how they play in improving progression free survival and overall survival through personalized cancer care. Not only do the genetic mutations frequently lend themselves to a targeted therapy but they can provide broader information about DNA mismatch repair (MMR), microsatellite instability (MSI), and the tumor mutational burden (TMB). MMR is a strand specific system for recognizing and repairing erroneous insertion, deletion, and misincorporation of nucleotides that can arise during DNA replication, as well as repairing some forms of DNA damage that can occur from toxin and radiation exposure. MSI is the condition within the cancer of genetic hypermutability (predisposition to mutation) that results from the impaired DNA mismatch repair (MMR) system. The presence of MSI accounts for the multiple cell lines that comprise every cancer. The DNA mutations lead to the production of abnormal proteins and provides phenotypic evidence that MMR is not functioning normally. The more abnormal the proteins produced are, the higher the TMB is and the easier it will be for the immune system to identify and attack the cancer cells. This correlates to a better clinical response to immunotherapy assuming that the immune system's infrastructure and biochemistry are still intact [141–146].

## 17. Hyperthermia

A useful adjunctive therapy, which is not a strategy unto itself, but works well with those described above, is hyperthermia. Hyperthermia can be used systemically, for example, with a far infrared sauna, locally with specialized microwave equipment, or intralesionally with radio frequency ablation. The goal of hyperthermia therapy is to raise the temperature of the cancer to as close to 107°F or 42°C as is possible without cooking healthy tissues or the patient. At those temperatures it can directly kill or weaken tumor cells by inducing apoptosis, with limited adverse effects on healthy cells. Due to their abnormal genetics, tumor cell defenses are compromised and, when coupled with their disorganized and compact vascular structure, have difficulty dissipating heat making them susceptible to this directed damage. As a secondary effect, if the cancerous cells do not die outright, they may become more susceptible to targeted radiation therapy and/or chemotherapeutic strategies. Tumor cell death caused by hyperthermia treatment induces the release of heat shock proteins (HSP). HSP70, in particular, when released into the extracellular milieu, can act simultaneously as an immune target due to its ability to chaperone antigenic peptides and also act as a maturation signal for nearby dendritic cells (DC), thereby inducing DCs to cross-present antigens to cytotoxic CD8+ T cells and natural killer cells. HSP can also act independently from associated peptides, stimulating the innate arm of the immune system [147–156].

## 18. Some Other Office Based Anticancer Strategies

There are other office based anticancer strategies such as those based on classical herbalism, traditional Chinese medicine, Ayurvedic medicine, western naturopathy, and homeopathy. These strategies can be very effective, but require years of study in specialized programs and are beyond the scope of this article. Functional medicine is a systems biology–based biochemical approach that focuses on identifying and addressing the “root cause” of disease and is not a treatment strategy unto itself. For example, immune dysfunction will result from poor nutrition, infection, toxins, trauma, and/or stress, and each must be investigated and addressed along with the specific immune dysfunction in order to reconstitute an effective immune response. Each symptom must be considered from a differential diagnosis perspective as it may prove be one of many contributing factors to an individual's illness.

## 19. Conclusion

Cancer, as a disease of our time, has been described as a “wound that does not heal” and thus has a multitude of biochemical dysfunctions at its core. However, in order for these genetically and phenotypically abnormal cells to survive and thrive, the watchdog of the body, the immune system, must itself be suffering from a number of serious areas of structural and functional damage. The therapies above are integrative and nonexclusive with each other. However, more aggressive cytotoxic strategies, i.e., full dose chemotherapy and radiation, can render many of these strategies null and void. When treating cancer, the physician and patient should make the decisions together, since the stakes can be no higher, using the best information available at the time, and then never look back. When treating cancer there is but one direction to go and that is forward, striving for ever increasing improvements in the quality and quantity of life with the hope of achieving a durable remission [157–160].

Research is needed to codify the algorithm necessary for applying the best therapeutic strategy given the dynamics of the patient's biochemistry and immunology at any given point in their treatment.
